# Correlation between obstructive sleep apnea and hypoperfusion in patients with acute cerebral infarction

**DOI:** 10.3389/fneur.2024.1363053

**Published:** 2024-04-08

**Authors:** Yi Zhou, Xiaomei Jin, Xiaorong Liu, Jiafan Tang, Liyan Song, Yu Zhu, Wanqing Zhai, Xianhui Wang

**Affiliations:** Department of Neurology, First People’s Hospital of Taicang, Taicang City, Jiangsu Province, China

**Keywords:** obstructive sleep apnea, apnea-hypopnea index, mean pulse oxygen, hypoperfusion, acute cerebral infarction

## Abstract

**Purpose:**

To explore the relationship between obstructive sleep apnea (OSA) and hypoperfusion during ultra-early acute cerebral infarction.

**Patients and methods:**

Data were retrospectively collected from patients admitted to our hospital with acute cerebral infarction between January 2020 and January 2022, who underwent comprehensive whole-brain computed tomography perfusion imaging and angiography examinations within 6 h of onset. The F-stroke software automatically assessed and obtained relevant data (Tmax). The patients underwent an initial screening for sleep apnea. Based on their Apnea-Hypopnea Index (AHI), patients were categorized into an AHI ≤15 (*n* = 22) or AHI >15 (*n* = 25) group. The pairwise difference of the time-to-maximum of the residue function (Tmax) > 6 s volume was compared, and the correlation between AHI, mean pulse oxygen saturation (SpO2), oxygen desaturation index (ODI), percentage of time with oxygen saturation < 90% (T90%), and the Tmax >6 s volume was analyzed.

**Results:**

The Tmax >6 s volume in the AHI > 15 group was significantly larger than that in the AHI ≤ 15 group [109 (62–157) vs. 59 (21–106) mL, *p* = 0.013]. Spearman’s correlation analysis revealed Tmax >6 s volume was significantly correlated with AHI, mean SpO2, ODI, and T90% in the AHI > 15 group, however, no significant correlations were observed in the AHI ≤ 15 group. Controlling for the site of occlusion and Multiphase CT angiography (mCTA) score, AHI (β = 0.919, *p* < 0.001), mean SpO2 (β = −0.460, *p* = 0.031), ODI (β = 0.467, *p* = 0.032), and T90% (β =0.478, *p* = 0.026) remained associated with early hypoperfusion in the AHI > 15 group.

**Conclusion:**

In patients with acute cerebral infarction and AHI > 15, AHI, mean SpO2, ODI and T90% were associated with early hypoperfusion. However, no such relationship exists among patients with AHI ≤ 15.

## 1 Introduction

Ischemic stroke is a major type of stroke, characterized by a high risk of disability and mortality (1, 2). Early thrombolysis and endovascular intervention are the most effective treatment strategies for cerebral infarction (3). Over the past 2 decades, perfusion mismatch ratio in computed tomography (CT) has been widely applied to assess ischemic tissue in patients with stroke. Quantitative parameters of CT perfusion (CTP) can visually reflect the cerebral blood flow perfusion of ischemic brain tissue (4). Time-to-maximum of the residue function (Tmax) is a highly responsive parameter for identifying hypoperfusion areas in perfusion imaging. The higher Tmax threshold reflects the lower the hypoperfusion volume of brain tissue, specifically, a threshold of Tmax >6 s has been used to assess severe hypoperfusion in tissue windows related to acute ischemic stroke (5, 6). It can accurately detect the penumbra (7). Studies have reported that Tmax strata proportions can predict neurological deterioration in patients with ischemic stroke (8, 9). However, few studies have reported the related influencing factors of volume of Tmax>6 s.

Obstructive sleep apnea (OSA) syndrome is characterized by repeated collapse of the upper airway, significantly reducing (hypopnea) or completely ceasing (apnea) airflow, resulting in episodic oxygen desaturation, excessive sympathetic activation, and sleep fragmentation (10). Studies have observed that OSA is associated with neurofunctional deterioration and increased mortality in patients with acute ischemic stroke (11). Severe OSA is a risk factor for stroke recurrence, potentially linked to higher stroke mortality, while continuous positive airway pressure (CPAP) can improve stroke prognosis (12, 13). Additionally, studies indicate that patients with OSA who receive intravenous recombinant tissue-type plasminogen activator (rt-PA) thrombolysis require prolonged hospitalization and recovery times (14). Neuroimaging studies have revealed OSA-associated brain structural changes. For example, patients with OSA have a heightened risk of disruption to their structural brain network (15). Moderate to severe OSA is independently associated with white matter change; however, mild OSA does not seem to influence the occurrence of white matter change in middle-aged and older individuals (16). A cohort study revealed a correlation between hypoxia and subcortical, hippocampal, and frontal gray matter atrophy in patients with OSA (17). However, currently, no research has reported on the relationship between OSA and early brain perfusion imaging abnormalities in patients with cerebral infarction. Therefore, the primary aim of this study was to investigate the relationship between specific risk factors of OSA and early cerebral hypoperfusion in patients with acute cerebral infarction.

## 2 Materials and methods

### 2.1 Study participants

This study retrospectively collected the data of patients with acute cerebral infarction who underwent the green channel process between 2020 and 2022. The inclusion criteria were: (1) Patients diagnosed with acute cerebral infarction according to the American Stroke Association diagnostic criteria; (2) Patients who underwent computed tomography perfusion imaging (CTP) and computed tomography angiography (CTA) examinations using a CT scanner (Philips, Netherlands) within 6 h of symptom onset. The F-stroke software (Neuroblem, Version 1.0.23) was used to obtain the perfusion volume with Tmax >6 s; and (3) Portable sleep respiratory monitoring was conducted within 1 week of stroke onset, with a 24-h prohibition on smoking, alcohol consumption, and the use of sedatives or sleep-inducing medications prior to the examination. The exclusion criteria were: (1) Patients who did not undergo CTA and CTP examinations within 6 h of stroke onset or did not complete the initial sleep respiratory screening within 1 week; (2) Patients with stroke history, a National Institutes of Health Stroke Scale (NIHSS) score of <4, and posterior circulation infarction; and (3) Patients with incomplete baseline data. The study was approved by the Ethics Committee of The First People’s Hospital of Taicang. The participants provided written informed consent to participate in this study.

### 2.2 Image acquisition and scale evaluation

All patients underwent the green channel process of ischemic stroke, and completed the computed tomography perfusion imaging (CTP) and computed tomography angiography (CTA) scan (Philips, Netherlands). CTA scanning was performed from the skull base to the top of the skull. The layer thickness was 0.625 mm. CTP scan was placed in a supine position with the head placed in the middle and fixed on a wedge sponge head rack. The images were constructed every 5 mm thick layer. The scan began after 5 s of contrast agent injection, 25 exposures were performed, and once for 2 s. All of the original scanned images were transferred to the workstation, and cerebral blood flow velocity (CBF), volume of Tmax>6S were identified by the F-stroke software. We evaluated collateral circulation by Multiphase CT angiography collateral (mCTA) score, firstly determined which hemisphere was affected by the image of CTP, and then assessed the statuses of collateral circulation using mCTA score on a scale of 0–5 according to the system described by Menon (18). We used apnealink air portable sleep monitor (ResMed, Australia) to detected sleep breathing parameters including nasal airflow, snore, saturation of pulse oxygen and respiration, and monitoring time > 7 h. Smoking, drinking, and sleeping drugs are prohibited 24 h before the examination. All brain MR examinations were performed on a 3-Tesla MR Unit (Siemens, Germany) during hospitalization. Neurologists rating the diffusion-weighted imaging, and scored the ASPECT blind to the patient’s disease information (19).

### 2.3 General data collection

General patient information, results of hematological tests, perfusion imaging parameters, and other clinical data were collected. Forty-seven patients were finally included in the study. General clinical information collected included age, sex, smoking status, medical history (hypertension, diabetes, and atrial fibrillation), admission systolic and diastolic blood pressures, random blood glucose concentration on admission, red blood cell count, hemoglobin concentration, platelet count, low-density lipoprotein levels, the NIHSS score, the diffusion-weighted imaging (DWI)-Alberta Stroke Program Early CT Score (ASPECTS), and the Multiphase CT angiography (mCTA) score. Sleep-related parameters, including the apnea-hypopnea index (AHI), mean pulse oxygen saturation (SpO2), oxygen desaturation index (ODI), and the percentage of time with oxygen saturation < 90% (T90%) were collected using a portable sleep respiratory monitor (Philips, Model: Alice Night One). The ODI was defined as the frequency of dips in SpO2 ≥ 3% per hour of sleep. The history of OSA was screened by STOP-Bang questionnaire. Two experienced neurologists assessed the NIHSS score, mCTA score, STOP-Bang score, and DWI-ASPECT score for all patients. Patients were categorized into two groups based on their AHI: ≤15 (*n* = 25) and > 15 (*n* = 22). Patients were divided into two groups: low risk of OSA (STOP-Bang<3) and high risk of OSA (STOP-Bang≥3).

### 2.4 Statistical analysis

Data were analyzed using SPSS software version 26.0 (Armonk, NY: IBM Corp). Continuous variables are presented as mean [standard deviation (SD)] or median (25th–75th percentile), and categorical variables are presented as percentages. The Shapiro–Wilk test was used for normality testing. A *t*-test was used for between-group comparisons for normally distributed data, while non-normally distributed data were analyzed using non-parametric tests. The chi-square test was used for categorical variables. Spearman’s correlation and linear regression analyses explored the relationship between AHI, mean SpO2, ODI, T90%, and the Tmax >6 s volume. The significance level was set at *p* < 0.05.

## 3 Results

### 3.1 Comparison of general and baseline characteristics between groups

Patients in the AHI > 15 group had higher body mass index (BMI), ODI and T90%, each *p* < 0.001, but lower mean SpO2 (*p* = 0.001). Patients with AHI > 15 were more likely to have larger Tmax >6 s volume [109(62–157) vs. 59 (21–106) mL, *p* = 0.013], larger CBF < 30% volume [24 (15–72) vs. 18 (6–27), *p* = 0.032], and lower mCTA scores [2 (1–3) vs. 3 (2–4), *p* = 0.013]. No significant differences were observed between groups in age, sex, nor proportion of patients with hypertension, diabetes, and atrial fibrillation. Additionally, no significant differences existed between the groups in admission systolic blood pressure, diastolic blood pressure, red blood cell count, hemoglobin concentration, platelet count, random blood glucose concentration, and low-density lipoprotein levels. Moreover, the NIHSS score and DWI-ASPECTS after admission, cerebrovascular stenosis, site of intracranial artery occlusion, and mismatch ratio did not significantly differ between the groups (Table 1).

**Table 1:** Baseline characteristics of the two groups.

Variables	AHI ≤ 15 (*n* = 25)	AHI > 15 (*n* = 22)	*p* value
Age (years)	71.84 ± 15.40	68.32 ± 12.72	0.401
Sex (female, *n*, %)	12 (48.0%)	6 (27.3%)	0.145
BMI (Kg/m^2^)	22.31 ± 1.69	25.42 ± 1.99	<0.001
Smoking (*n*, %)	6 (24.0%)	7 (31.8%)	0.550
Time to onset (min)	213 (123–261)	186 (98–299)	0.876
Diabetes (*n*, %)	5 (20.0%)	6 (27.3%)	0.557
Atrial fibrillation (*n*, %)	5 (20.0%)	7 (31.8%)	0.354
Hypertension (*n*, %)	12 (48.0%)	12 (54.5%)	0.654
SBP on admission (mmHg)	146.96 ± 20.02	152.82 ± 16.86	0.163
DBP on admission (mmHg)	82.80 ± 15.15	88.64 ± 12.74	0.163
Red blood cell (X 10^12^/L)	6.46 ± 1.71	6.54 ± 1.38	0.861
Hemoglobin (g/L)	136.44 ± 11.34	140.18 ± 13.50	0.307
Blood platelet (X 10^9^/L)	158.92 ± 40.18	167.41 ± 41.27	0.479
Admission glucose (mmol/L)	5.58 ± 1.35	6.38 ± 1.43	0.054
LDL (mmol/L)	3.30 ± 0.89	3.59 ± 0.61	0.206
Cerebrovascular stenosis (*n*, %)	8 (32.0%)	9 (42.9%)	0.447
Site of intracranial artery occlusion			
ICA (*n*, %)	3 (12.0%)	5 (22.7%)	0.624
MCA (*n*, %)	5 (20.0%)	4 (18.2%)	
CBF < 30% volume (mL)	18 (6–27)	24 (15–72)	0.032
Tmax>6S volume (mL)	59 (21–106)	109 (62–157)	0.013
Mismatch ratio	2.53 (1.14–5.38)	1.53 (0.87–3.76)	0.316
IVT (*n*, %)	9 (36.0%)	7 (31.8%)	0.953
EVT (*n*, %)	2 (8.0%)	2 (9.1%)	
Mean SpO2 (%)	96.60 ± 1.58	92.09 ± 3.82	0.001
ODI (events/h)	4 (2–6)	23 (20–28)	<0.001
T90% (%)	3.12 ± 1.09	20.14 ± 6.18	<0.001
mCTA scores	3 (2–4)	2 (1–3)	0.013
NIHSS scores	10 (6–16)	13 (5–18)	0.460
DWI-ASPECT scores	4 (2–6)	4 (2–7)	0.644

AHI, Apnea-hypopnea index; BMI, Body mass index; SBP, Systolic blood pressure; DBP, Diastolic blood pressure; LDL, Low density lipoprotein; MCA, Middle cerebral artery; ICA, Internal carotid artery; CBF, Cerebral blood flow; IVT, Intravenous alteplase; EVT, Endovascular therapy; SpO2, Pulse oxygen saturation; ODI, Oxygen desaturation index; T90%, Percentage of time with oxygen saturation < 90%; mCTA, Multiphase CT angiography; NIHSS, National Institutes of Health Stroke Scale; and DWI-ASPECT, Diffusion-weighted imaging-Alberta Stroke Program Early CT.

### 3.2 Comparison of hypoperfusion volume between low and high risk of OSA

**Figure 1:**
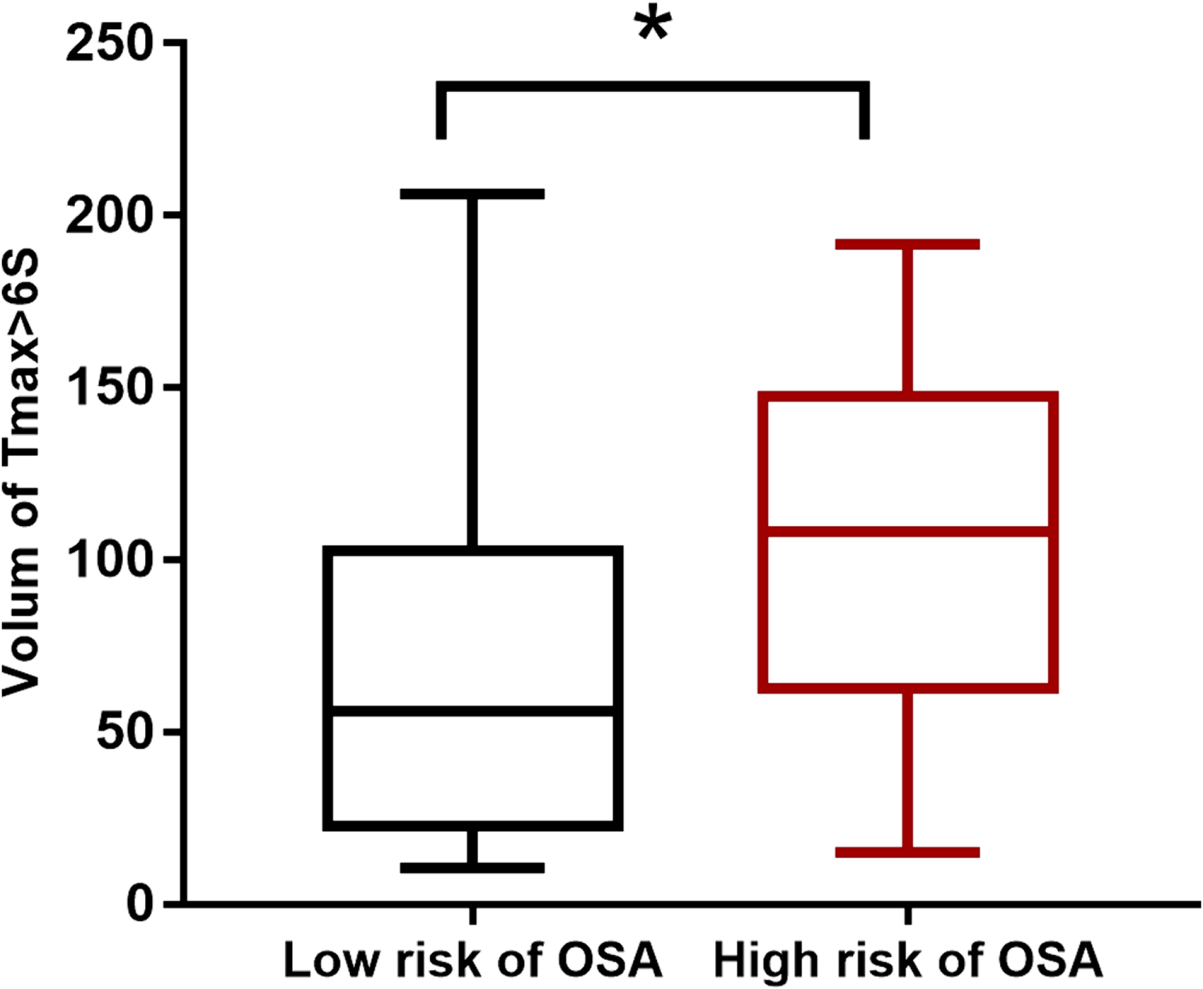
Comparison of hypoperfusion volume between low and high risk of OSA (^*^p < 0.05).

The comparison of the Tmax >6 s volume between low and high risk of OSA groups revealed a significant difference (*p* = 0.030). The Tmax >6 s volume in the high risk of OSA group was 108 (62–147) mL, while it was 56 (22–102) mL in the low risk of OSA group (Figure 1).

### 3.3 Correlation between AHI and hypoperfusion

**Figure 2:**
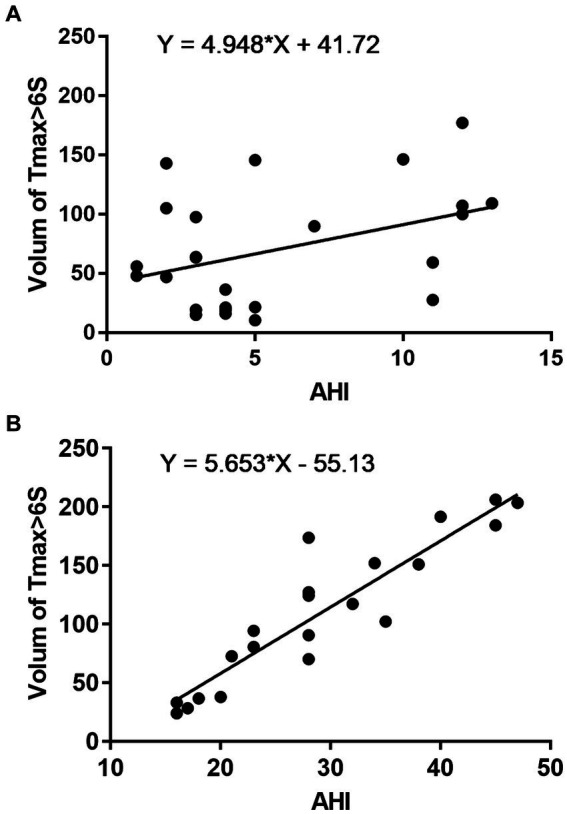
Correlation between AHI and hypoperfusion. AHI ≤ 15 **(A)** and AHI > 15 **(B)**.

Spearman’s correlation analysis revealed that, in the AHI ≤ 15 group, no significant correlation existed between AHI and the Tmax >6 s volume (*r*_s_ = 0.277, *p* = 0.180; Figure 2A). However, in the AHI > 15 group, a positive correlation was observed between AHI and the Tmax >6 s volume (*r*_s_ = 0.903, *p* < 0.001; Figure 2B).

### 3.4 Correlations between mean SpO2, ODI, T90%, and hypoperfusion

**Figure 3:**
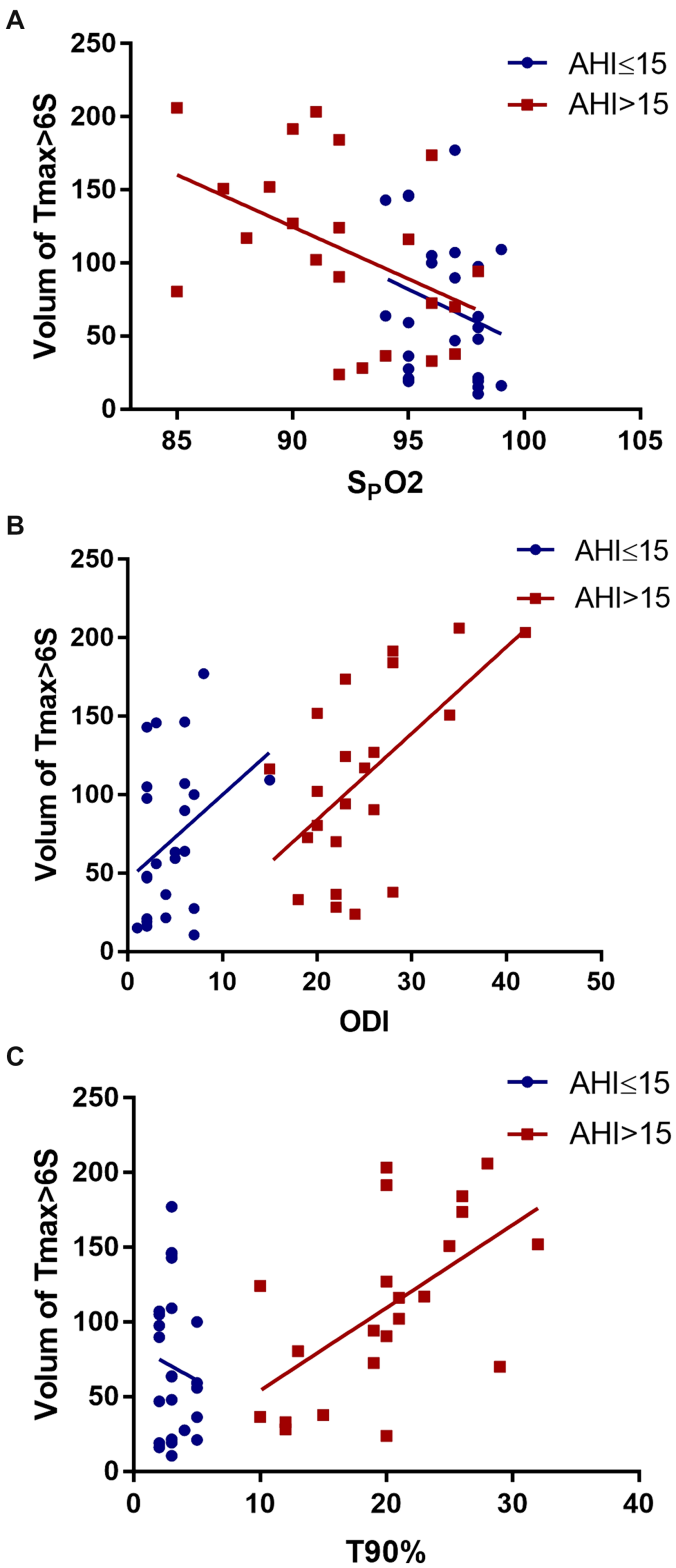
Correlation between mean SpO2 **(A)**, ODI **(B)**, T90% **(C)**, and hypoperfusion.

Spearman’s correlation analysis revealed no significant correlation between SpO2 (*r*_s_ = −0.291, *p* = 0.240), ODI (*r*_s_ = 0.283, *p* = 0.170), T90% (*r*_s_ =0.025, *p* = 0.905), and the Tmax >6 s volume in the AHI ≤ 15 group. In contrast, in the AHI > 15 group, a negative correlation was observed between mean SpO2 and the Tmax >6 s volume (*r*_s_ = −0.518, *p* = 0.014). ODI (*r*_s_ = 0.534, *p* = 0.010) and T90% (*r*_s_ = 0.565, *p* = 0.006) were also related with Tmax >6 s volume (Figure 3).

### 3.5 Linear regression analysis

Multiple linear regression analysis was conducted with Tmax >6 s volume as the dependent variable, AHI, mean SpO2, ODI, and T90% as the independent variables, respectively, controlling for the site of occlusion and mCTA score in order to eliminate confounder effects. The results revealed a significant correlation between AHI (β = 0.919, *p* < 0.001), SpO2 (β = −0.460, *p* = 0.031), ODI (β = 0.467, *p* = 0.032), T90% (β = 0.478, *p* = 0.026), and the Tmax >6 s volume (Table 2).

**Table 2:** Multiple linear regression analysis.

Variables	Unstandardized coefficients		Standardized coefficients	*T* value	*p* value	95% CI
	B	SD	β			
AHI	5.653	0.542	0.919	10.436	<0.001	4.523–6.783
Mean SpO2 (%)	−7.121	3.077	−0.460	−2.314	0.031	−13.540 to −0.702
ODI (events/h)	4.482	1.925	0.467	2.328	0.032	0.437–8.527
T90%	4.575	1.882	0.478	2.431	0.026	0.622–8.529

AHI, Apnea-hypopnea index; SpO2, Pulse oxygen saturation; ODI, Oxygen desaturation index; T90%, Percentage of time with oxygen saturation < 90%; CI, Confidence interval.

## 4 Discussion

The study findings indicated that, in patients with acute cerebral infarction and AHI ≤ 15, no significant correlation existed between the Tmax >6 s volume and AHI, mean SpO2, ODI, or T90%. Nevertheless, in patients with AHI > 15, a positive correlation existed between the Tmax >6 s volume and AHI, ODI, T90%. Conversely, mean SpO2 negatively correlated with the Tmax >6 s volume for this group of patients. This suggests that moderate-to-severe OSA may significantly impact hypoperfusion in patients with acute cerebral infarction, whereas mild OSA seems insignificant.

Perfusion imaging provides estimates of cerebral hemodynamics, with Tmax being one of the most used evaluation parameters. Clinically, the Tmax >6 s area is an area of inadequate perfusion, and irreversible brain tissue damage occurs if reperfusion is not restored in this region (6). Studies have revealed that the baseline Tmax >6 s volume significantly correlates with the volume of infarction 27 h after stroke onset (20), indicating a close relationship between early hypoperfusion and the final infarction volume. This study revealed a positive correlation between the AHI and Tmax >6 s volume in patients with acute cerebral infarction and AHI > 15, suggesting an influence of OSA on cerebral perfusion. Oxygen metabolism is pivotal for determining post-stroke tissue vitality (21), and research has indicated that severe nighttime hypoxemia (oxygen saturation < 90% for >10% of the night) in older males increase the risk of stroke by 1.8 times (22). Hypoperfusion in patients with OSA is related to sleep fragmentation and hypoxia (23). Cerebral venous oxygen content is associated with the volume of hypoperfusion (Tmax >4 s) (24). In the current study, we observed negative correlation between mean SpO2 and the Tmax >6 s volume, moreover, ODI and T90% positively affected Tmax>6 s volume in patients with OSA. It furtherly emphasized the association between hypoxia and hypoperfusion. Some studies have suggested that severely hypoperfused regions account for a higher proportion of the brain tissue with poor collateral circulation in patients with large vessel occlusion (25). We conducted multiple linear regression analysis in order to adjust the underlying confounding factors, including collateral circulation and site of occlusion, the association was also observed. This results furtherly confirmed the influence of severe OSA on hypoperfusion. Multiple researches have reported a bidirectional relationship between OSA and stroke: on the one hand, it is a risk factor for stroke, on the other hand, it is also a consequence (26, 27). The AHI was detected after the onset in the current study, it could not reflect the previous severity, and this made it difficult to determine the causal relationship between OSA and hypoperfusion. STOP-Bang questionnaire is a reliable screening tool for identify the risk of OSA, STOP-Bang score ≥ 3 was defined as high risk of OSA (28). Our study found patients pre-existing high risk of OSA got larger Tmax>6 s volume, therefore, we speculated that OSA may influence hypoperfusion. Previous studies have not explored the impact of OSA on cerebral collateral circulation, we observed a noteworthy disparity in the collateral circulation mCTA scores between the AHI ≤ 15 and > 15 groups. However, due to the small sample size of our study, further researches are demanded to explore the effect of OSA on collateral circulation. Prior studies have indicated that high AHI in patients with OSA is an independent risk factor affecting carotid intima-media thickness (29); nonetheless, research on the association between OSA and carotid and cerebral vascular stenosis is limited. This study did not reveal significant differences in vascular stenosis between groups categorized by AHI. In our study, the ASPECT score was very low, it probably because that we performed magnetic resonance diffusion-weighted imaging (DWI) assessment, and the patients completed the examination several days after admission to hospital.

The relationship between OSA and cerebral infarction remains controversial. Studies have suggested that OSA can lead to an adverse prognosis in cerebral infarction, impair brain function, exacerbate cognitive dysfunction, and increase mortality risk (30–33). The main mechanisms underlying these adverse outcomes include chronic intermittent hypoxia, sympathetic activation, changes in cerebral blood flow, oxidative stress, systemic inflammation, hypercoagulability, and endothelial dysfunction (34, 35). However, recent research has proposed the possibility of a causal relationship between OSA and stroke being non-existent. This study overcame some limitations of previous research by adjusting for confounding factors; however, it did not classify OSA severity (36). Additionally, other studies have suggested that patients with acute cerebral infarction and OSA may experience less severe neurological impairment and lower mortality than those without obstructive sleep apnea (37, 38). This protective effect could be attributed to the intermittent hypoxia resulting from recurrent apnea and hypoventilation in patients with sleep apnea, leading to ischemic adaptation. Therefore, the impact of OSA on the prognosis of patients with cerebral infarction remains debatable. Our study conducted separate statistical analyses based on OSA severity and observed no significant difference of NIHSS scores between the two groups. Furthermore, positive correlations were observed between the AHI, ODI, T90% and Tmax >6 s volume, and negative associations between mean SpO2 and Tmax >6 s volume in the AHI > 15 group. Therefore, we infer that the severity of OSA and hypoxia are related to early cerebral hypoperfusion in patients with acute cerebral infarction.

This study has some limitations. Firstly, it was an exploratory study with a small sample size. Secondly, the assessment of sleep apnea events in this study utilized initial screening. While less accurate than polysomnography for diagnosing OSA, this approach remains a relatively good and cost-effective alternative and is suitable for patients with serious OSA. Additionally, stroke can aggravate OSA, however, all patients in this study underwent initial screening after experiencing cerebral infarction, which could exacerbate sleep apnea, possibly resulting in higher AHI scores. OSA and stroke often share common risk factors which may cause confounding factors interference, we hope that further prospective study will make clear our results. Also, the study did not include patients with posterior circulation infarction, which might have introduced some selection bias. Lastly, patients with cerebral infarction history were excluded due to their increased hypoperfusion volumes; therefore, the study results might not apply to such patients.

## 5 Conclusion

In conclusion, this study suggests that OSA influences early cerebral hypoperfusion in patients with acute cerebral infarction. The AHI, mean SpO2 levels, ODI and T90% significantly correlated with poor cerebral perfusion in patients with moderate-to-severe OSA. However, conducting additional large-scale prospective studies to validate and substantiate these findings is essential.

## Data availability statement

The raw data supporting the conclusions of this article will be made available by the authors, without undue reservation.

## Ethics statement

The studies involving humans were approved by Medical ethics committee, The First People’s Hospital of Taicang. The studies were conducted in accordance with the local legislation and institutional requirements. The participants provided their written informed consent to participate in this study.

## Author contributions

YiZ: Data curation, Writing – original draft. XJ: Data curation, Investigation, Writing – original draft. XL: Data curation, Investigation, Writing – original draft. JT: Investigation, Methodology, Validation, Writing – original draft. LS: Data curation, Investigation, Writing – original draft. YuZ: Data curation, Software, Writing – original draft. WZ: Project administration, Writing – original draft. XW: Funding acquisition, Methodology, Supervision, Writing – review & editing, Formal analysis, Validation.
